# A Comparison of Ultra-Widefield Imaging Quality Obtained with Zeiss Clarus and Optos for Virtual Medical Retina Services

**DOI:** 10.3390/jcm14103270

**Published:** 2025-05-08

**Authors:** Matthew Azzopardi, Sneha Gridhar, Chrysanthi Tsika, Georgios Koutsocheras, Michail Katzakis, Bahar Demir, Waheeda Rahman, Ling Zhi Heng, Yu Jeat Chong, Abison Logeswaran

**Affiliations:** 1 Moorfields Eye Hospital NHS Foundation Trust, City Road, London EC1V 2PD, UK; matthew.azzopardi.14@um.edu.mt (M.A.); sneha.giridhar@nhs.net (S.G.); chrysanthi.tsika@nhs.net (C.T.); g.koutsocheras@nhs.net (G.K.); michail.katzakis@nhs.net (M.K.); bahar.demir@nhs.net (B.D.); w.rahman@nhs.net (W.R.); ling.heng@nhs.net (L.Z.H.); 2 Birmingham and Midland Eye Centre, Birmingham B18 7QH, UK; yujeat.chong07@alumni.imperial.ac.uk

**Keywords:** medical retina virtual clinic, ultrawide-field imaging, Optos, Zeiss Clarus

## Abstract

**Background:** Virtual clinics (VCs) have proven to be an effective solution for the increasing strain on Medical Retina (MR) services, although imaging quality issues (IQIs) persist. Our aim was to compare the quality of two ultra-wide-field (UWF) imaging modalities (Optos and Clarus) in real-world MR-VC settings. **Methods:** We conducted a real-world, prospective study. Data were collected from 6 Moorfields NHS Trust MR-VCs between September and October 2024. We obtained patient demographics and characteristics, primary diagnosis, UWF imaging types and images obtained, and follow-up outcomes. **Results:** Optos (California RG/RGB, and Monaco) was used for 56.7% (*n* = 152) and Zeiss Clarus 500 for 43.3% (*n* = 116) of the total cohort (*n* = 268). No statistically significant difference (*p* = 0.14) was found between the two in terms of the rates of IQIs. FAF (*p* = 0.001) acquisition was significantly higher when Optos was used. Of the patients affected by IQIs, 10 were examined in a face-to-face clinic (F2FC). No difference in IQI rates was observed when pathology-related poor image quality was considered (*p* = 0.561). A significantly (*p* = 0.001) higher rate of F2F follow-ups was found for red-flag pathologies and unexplained vision loss, with a statistically significantly higher rate of virtual follow-ups for non-red-flag pathologies (*p* = 0.001). **Conclusions:** A total of 7.5% of the clinical decisions were impacted by IQIs; 11.1% of F2FC follow-ups. Neither UWF imaging modality type was inferior in terms of IQI rates. FAF acquisition was higher with Optos, likely representing greater user-dependency for Clarus. The outcomes were not influenced by FAF acquisition, indicating that routine acquisition is not required in MR-VCs and instead should be obtained when clinically required.

## 1. Introduction

Ophthalmology is the busiest hospital outpatient specialty in England, with over 8.9 million attendances recorded for the 2023–2024 financial year, accounting for 8.56% of all outpatient attendances throughout English NHS Hospitals and English NHS-commissioned activity in the independent sector [1].

In recent years, growing strain on hospital ophthalmic services has resulted in significant capacity challenges, with a substantial proportion of patients across the United Kingdom experiencing delays in appointments or being lost to follow-up [2,3]. A 2017 British Ophthalmological Surveillance Unit (BOSU) study reported that between 15 and 22 patients a month suffered preventable permanent, severe sight loss due to health service-initiated delays [4]. Escalating pressures, intensified by the COVID-19 pandemic, precipitated significant paradigm shifts in the delivery of healthcare, most notably marked by the growing adoption of virtual or remote assessments. This innovative model of service provision has been widely adopted across numerous hospital-based ophthalmic services, including medical retina (MR) services, one of the highest-volume ophthalmic subspecialties [3]. Virtual assessment clinics are commonly used to monitor stable, low-risk MR conditions, such as treated neovascular age-related macular degeneration (nAMD) and mild non-proliferative diabetic retinopathy (DR) [5,6,7], and for referral refinement [8,9,10].

These virtual clinics have been shown to improve service capacity and alleviate the pressure on hospital services [5,11], thus decreasing referral-to-treatment (RTT) waiting times. They also contribute to improving patient journey times [12] and patient outcomes [5] while also maintaining staff costs comparable to those of face-to-face clinics (F2FCs) [13].

However, significant issues have been raised regarding imaging quality. Retinal imaging in most MR virtual clinics typically consists of optical coherence tomography (OCT) and ‘ultra-widefield’ (UWF) imaging [5,7]. In recent years, the term UWF has been used loosely, and often interchangeably with ‘widefield’, by manufacturers and clinicians alike due to a lack of a clear definition. To address this, the International Widefield Imaging Study Group, an international panel of retinal imaging experts, proposed that the term ‘UWF’ should be used for images capturing a field of view (FoV) of approximately 110–200°, encompassing the retina beyond the anterior edge of the vortex vein ampulla up to the pars plana. ‘Widefield’ imaging, by contrast, refers to views extending to the posterior edge of the vortex vein ampulla (approximately 60–100° FoV), while ‘panretinal’ imaging denotes full 360° coverage from ora to ora [14].

Although non-mydriatic UWF imaging has been shown to be as reliable as dilated fundus examination—and offer much faster acquisition times [15,16]—virtual clinics’ efficiency remains contingent upon high-quality imaging. Poor image quality not only adversely affects clinical decision making but also increases unnecessary referrals to F2FCs. Indeed, a 2018 study by Kortuem et al. identified poor imaging quality as the commonest reason for F2FC follow-ups for patients examined at MR virtual clinics [9]. To date, however, there have been no studies directly comparing the image quality of UWF modalities or the clinical implications associated with these differences.

Our primary aim was to assess and compare the quality of UWF fundus imaging and fundal autofluorescence acquisition between two different imaging modalities (Zeiss Clarus 500 and Optos California/MonacoPro) in a real-world MR virtual service (MR-VS) setting. Our secondary aims were to describe the distribution of primary retinal pathologies reviewed and the main reasons for virtual follow-ups versus those held at F2FCs.

## 2. Materials and Methods

### 2.1. Study Design

This real-world, prospective study was conducted across 6 MR virtual clinic sites within Moorfields NHS Foundation Trust: Moorfields City Road, Moorfields Brent Cross, Moorfields Hoxton, Moorfields Stratford, Moorfields Ealing, and Moorfields Northwick Park. It was designed as a pragmatic service evaluation, intended to capture a real-world sample within the available clinical audit approval window. It was approved by the Moorfields Clinical Audit and Effectiveness Team and conducted in accordance with the tenets of the Declaration of Helsinki.

Data were entered into a secure database by clinicians conducting virtual clinic reviews, who comprised senior fellows and consultants, over 2 months in September and October 2024. Any uncertainties in clinical decision-making were consistently discussed with the supervising consultant. Data were inputted into a secure database by the clinicians performing the virtual clinic reviews, who were either senior fellows or consultants. Any uncertainties were always discussed with the consultant. Only cases in which clinical outcomes were influenced primarily by UWF imaging findings were included; OCT was part of the VC assessment, but it was not the focus of this analysis.

The data collected included demographics; virtual clinic visit details, including the site; primary diagnosis; type of visit (new versus follow-up); type of future follow-up outcome (virtual versus face-to-face) and time frame; primary reasons for follow-up; and type of UWF imaging modality used and whether FAF images were acquired. Primary reasons for follow-ups were categorised into reviews for clinical reasons alone; the presence of a clinically significant artefact; the presence of a clinically significant insufficient FoV; the presence of clinically significant poor image quality; and a need for further imaging beyond UWF and OCT. The term ‘red-flag pathology’ was used to refer to serious/potentially serious pathologies that would typically warrant earlier F2FC review, such as ocular melanoma, neovascularisation, and active choroidal neovascularisation.

### 2.2. Moorfields Medical Retina Virtual Clinic Pathway

**Figure 1:**
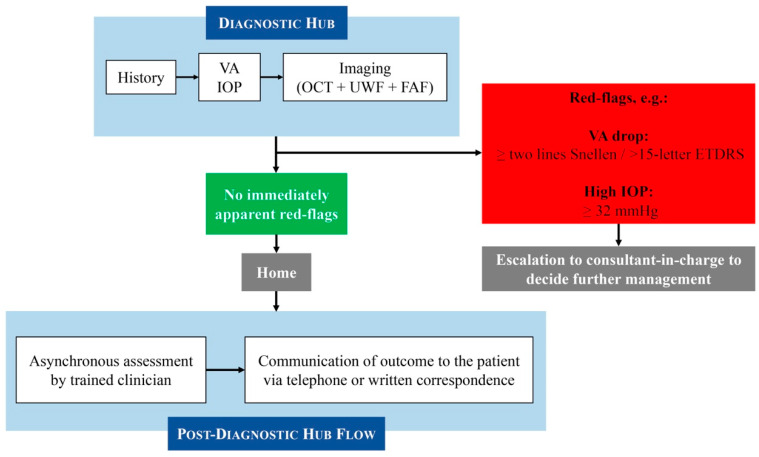
Moorfields Medical Retina virtual clinic pathway (at the time this study was conducted).

The structure of our MR-VS was previously described in [7]. In summary, the patients attend technician-led diagnostic hubs for assessment, which involves disease-specific history taking, measurement of visual acuity (VA) and intraocular pressure (IOP), and retinal imaging including OCT, UWF fundus photos, and fundus autofluorescence (FAF). All of these images and parameters are obtained by trained technicians following comprehensive protocols designed to minimise inter-operator variability. All of these clinical parameters and images are then assessed asynchronously at consultant-led clinics by trained clinicians (usually senior optometrists, senior fellows, or consultants), with disease-specific outcome pathways guided by established protocols. The diagnosis and management plan are then communicated to the patient through written correspondence or telephone consultation if urgent assessment or treatment, or further discussion, is required. In exceptional cases, patients are flagged and discussed with the consultant in charge at the virtual clinic. The consultant will then review the history and images and decide whether same-day clinic or casualty review is required. Figure 1 shows the overall Moorfields MR-VS pathway at the time this study was conducted. Whilst minor operational adjustments have been made to optimise flow since collecting the data, the core clinical pathway remains consistent.

### 2.3. Ultrawide-Field Imaging

The two main imaging modalities capable of obtaining UWF images (termed ‘UWF imaging’ in this study) used throughout the Moorfields MR-VS are the Zeiss Clarus 500 (Carl Zeiss Meditec AG, Oberkochen, Germany) and Optos California (Optos, Dunfermline, Scotland, UK).

With regard to site-specific imaging modality use, Zeiss Clarus 500 is the UWF imaging modality used at the Hoxton, Stratford, and Northwick Park sites, whilst City Road, Brent Cross, and Ealing sites use Optos California RG in most cases. In some cases, Optos MonacoPro is also used at the Ealing site, and Optos California RGB at City Road. For the purposes of this study, all Optos imaging systems have been grouped together as ‘Optos’, since they all have the same single-image FoV, clarity, and usability.

As per the Moorfields MR-VS imaging protocol, when Zeiss Clarus is used, 4-field colour and FAF photos should be taken, whilst for Optos, single-capture colour photos and FAF are required.

### 2.4. Imaging Quality Indices: Definitions and Examples

**Figure 2:**
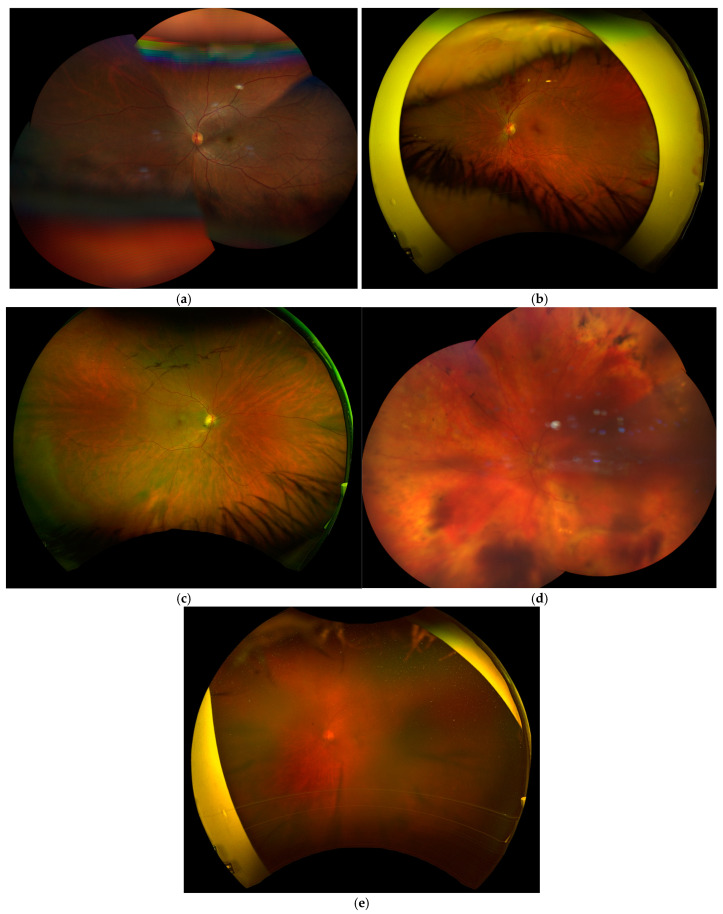
Example images from study patients: (**a**) insufficient field of view obtained using Clarus; (**b**) insufficient field of view obtained using Optos; (**c**) artefact in Clarus imaging; (**d**) artefact in Optos imaging; and (**e**) poor image quality obtained using Optos.

The definitions of the Imaging Quality Indices used in this study are shown in Table 1, along with the examples in Figure 2.

### 2.5. Statistical Analysis

Statistical analysis was conducted using SPSS 24.0 (IBM Corp, Armonk, NY, USA). Normality of the data was assessed using the Shapiro–Wilk test. A Fisher Exact test was performed to determine differences between nominal data, and a post hoc analysis was included when needed. For continuous data between two groups, Mann–Whitney test was used.

## 3. Results

### 3.1. Patient Demographics and Baseline Characteristics

A total of 268 patients were included in this study. The average age was 63 (SD ± 17), with the majority (*n* = 212; 79.1%) being follow-up patients. Optos was used in 56.7% (*n* = 152) of cases, whilst Clarus was used in 43.3% (*n* = 116) of cases. A full break-down of the baseline characteristics is shown in Table 2, alongside a comparison of the Optos and Clarus imaging subgroup characteristics.

### 3.2. Primary Retinal Diagnoses

The majority of patients reviewed through the MR-VS had diabetic retinopathy or maculopathy (DR/DM; 31.7%), AMD (14.9%), CSCR (13.8), or retinal vein occlusion (RVO; 10.8%). There were no statistically significant differences between primary diagnoses for the Optos and Clarus imaging subgroups (*p* > 0.05). Table 3 shows the full list of pathologies reviewed via the MR-VS over the study period.

A further breakdown of the vitreoretinal and ‘other’ pathologies reviewed is available in the Supplementary Material.

### 3.3. Primary Results

The vast majority of follow-ups were guided by clinical need alone (92.0% for Optos versus 93.1% for Clarus), including those brought to F2FCs (83.8%; *n* = 83).

**Figure 3:**
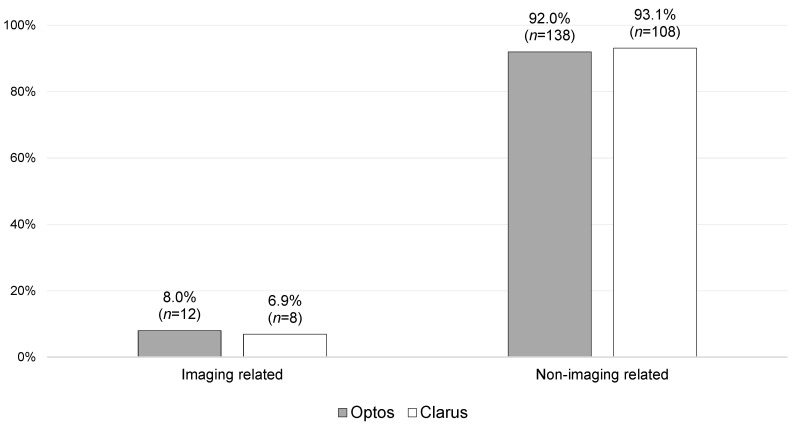
Primary reasons for follow-up stratified into imaging-related and non-imaging-related reasons, with a comparison of the Optos and Clarus sub-groups [*n* = 266; missing data = 2].

**Figure 4:**
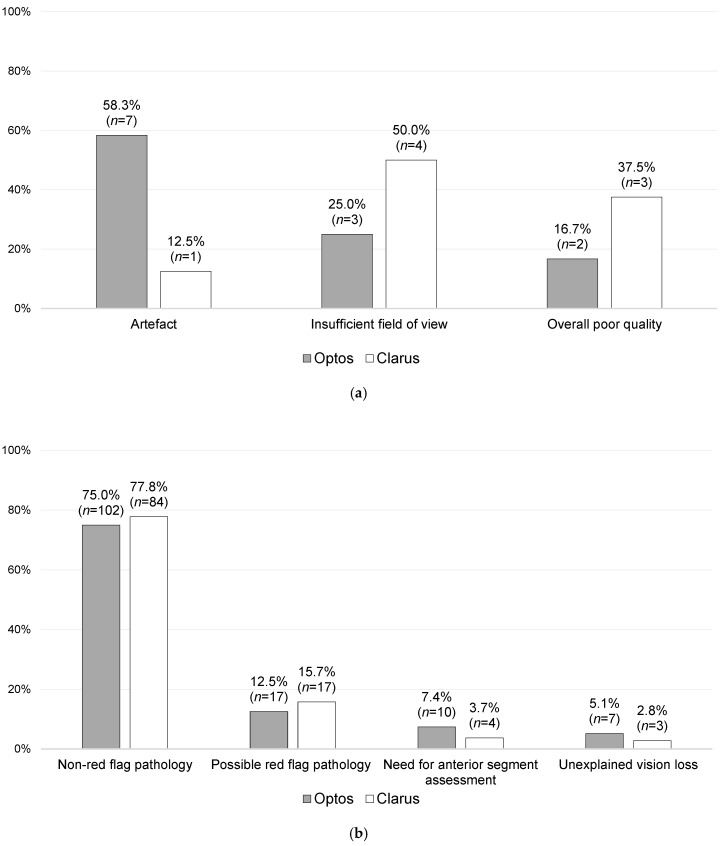
Further stratification of the primary reasons for follow-ups, with a comparison of the Optos and Clarus sub-groups: (**a**) imaging-quality-related primary reasons for follow-ups [*n* = 20], and (**b**) non-imaging-quality-related primary reasons for follow-ups (related to clinical need) [*n* = 244; missing data = 2].

Overall, no statistically significant difference (*p* = 0.228) was found between the Optos and Clarus imaging subgroups in terms of the primary reasons for outcomes. There were also no statistically significant differences found between Optos and Clarus when the imaging-related and non-imaging-related primary reasons for outcomes were stratified and compared, as shown in Figure 3 and Figure 4.

As shown in Figure 4a, 20 patients (7.5%) had follow-up outcomes primarily based on UWF-imaging-quality-related issues, of which there were 12 from the Optos imaging subgroup (8.0% of the Optos imaging patients) and 8 from the Clarus imaging subgroup (6.9% of the Clarus imaging patients). Of these 20 patients, 9 were followed up virtually (3 did not attend [DNA]), while 11 were brought to the F2FC (1 DNA), accounting for 11.1% of the overall F2FC follow-ups. Of these latter cases, 2.0% (*n* = 2) were referred to F2FCs due to artefacts, 4.0% (*n* = 4) were referred due to an insufficient FoV, and 5.1% (*n* = 5) were referred due to overall poor quality.

Amongst the 10 patients who subsequently visited an F2FC for a follow-up, 3 had a pathology that could explain the imaging quality issues: one had a dislocated intraocular lens (IOL), another patient had a head tremor secondary to Parkinson’s disease, and the last patient had vitreous haemorrhage. The remaining seven cases had no identifiable pathologies that could explain the impaired imaging quality. However, even after excluding the three cases with pathology-related poor image quality, no statistically significant difference was observed in the rate of imaging quality issues between Optos and Clarus imaging modalities (*p* = 0.561).

With regard to FAF acquisition, all the patients for whom Optos was used had FAF photos (100%; *n* = 152), whilst for Clarus, 77.6% had FAF photos (*n* = 90). This was found to be statistically significant (*p* = 0.001).

### 3.4. Secondary Results

**Figure 5:**
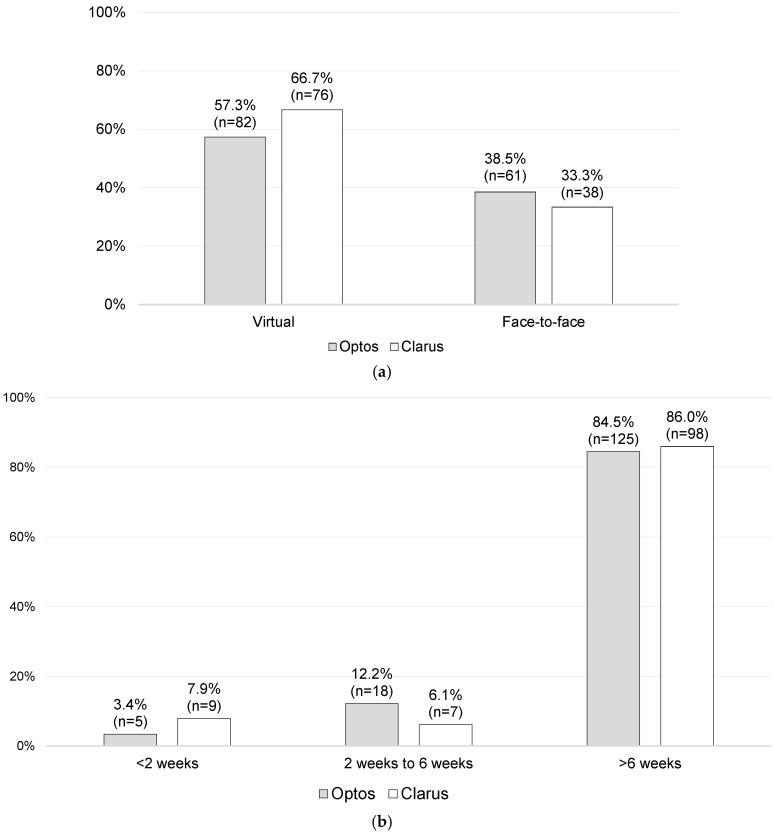
Follow-up data: (**a**) follow-up setting [*n* = 257; missing data = 11] and (**b**) follow-up period [*n* = 262; missing data = 6].

Of the overall cohort (*n* = 262; missing data = 6), 61.5% (*n* = 158) were followed up virtually, while the rest attended an F2FC follow-up appointment (38.5%; *n* = 99). No statistically significant differences were found between the Optos and Clarus imaging subgroups with regard to virtual versus F2FC follow-ups (*p* = 0.156). In total, 57.3% of the Optos imaging subgroup and 66.7% of the Clarus imaging subgroup were followed up virtually, whilst 38.5% and 33.3% of the Optos and Clarus imaging subgroups, respectively, were brought to F2FC for a follow-up, as shown in Figure 5a. The follow-up period intervals were also very similar, as shown in Figure 5b.

A total of 37.8% of the F2FC follow-ups were related to non-red-flag retinal pathologies, whilst 32.9% were related to a suspicion of a red-flag pathology. A statistically significantly higher rate of F2F follow-ups was found overall for red-flag pathologies (*p* = 0.001), anterior segment assessments (*p* = 0.001), and unexplained vision loss (*p* = 0.001). A statistically significantly higher rate of virtual follow-ups was found for non-red-flag pathologies (*p* = 0.001).

## 4. Discussion

To the best of our knowledge, this is the first real-world study comparing the imaging quality of the two main UWF imaging modalities in use in MR-VSs and the respective impacts on clinical decision-making and follow-ups. The cohort demographics were similar in both groups, and no statistically significant differences that could influence the results were noted. The only differing factor was the higher proportion of follow-up patients (versus new patients) in the Clarus subgroup. These patients may benefit from improved imaging due to technician familiarity with individual patient needs, thus potentially enhancing image acquisition consistency. However, as there was no significant difference in the overall imaging quality indices between modalities, this did not influence the main outcome. Similar to the findings in other published studies [6,7,17], the main diagnoses reviewed were DR and AMD, followed by retinal vein occlusion (RVO) and central serous chorioretinopathy (CSCR).

A total of 88.9% of the F2FC follow-ups post VC review were due to clinical need, with the majority being related to either non-red-flag retinal pathologies (37.8%) or a suspicion of a red-flag pathology (32.9%). A statistically significantly higher rate of F2F follow-ups was found for red-flag pathologies, alongside anterior segment assessments and reviews of unexplained vision loss (*p* = 0.001), whilst non-red-flag pathologies were predominantly followed up virtually (*p* = 0.001). This indicates that whilst routine/less urgent cases are being managed in MR-VSs, patients with red-flag pathologies and unexplained vision loss are correctly being brought to F2FCs for clinician review and further management.

Imaging quality issues such as artefacts, an insufficient FoV, and poor overall image quality were cited as the main determining factor for clinical outcomes in 7.5% of cases (20 patients). However, no statistically significant difference was found between the Optos and Clarus imaging modalities in terms of the rate of imaging quality issues (8.0% for Optos vs. 6.9% for Clarus; *p* = 0.14). Of these 20 patients, 11 were brought to an F2FC, representing 11.1% of all the patients who were followed up at F2FCs after their MR-VS appointment. This is quite significant, since an imaging-quality-issue-related F2FC bring-back rate of 1 in 10 undermines both the cost-efficiency and scope of VCs whilst also contributing to increased strain on F2FC capacity. Whilst significant efforts are being made to reduce outpatient DNA rates [18], which are currently reported to be around 15–22% in VCs [12,19], similar attention should be directed towards minimising bring-back rates due to suboptimal imaging quality. Both of these issues impact VC workflow efficiency and resource utilisation, and thus addressing them in parallel would significantly enhance MR-VS effectiveness.

There was also no significant effect of the type of imaging modality on clinical-requirement-based outcome decisions (uninfluenced by imaging quality) (*p* = 0.43). This indicates that neither UWF modality is inferior to the other with regard to both poor and good image quality and that having one over the other in an MR-VS is unlikely to affect clinical decision-making.

When comparing the two UWF imaging modalities and their use in the MR-VSs, there are some important differences that users should be aware of. The technical specifications of the two have been summarised in Table 4.

To ensure that MR-VSs run efficiently and as expected, systems engineering principles can be applied, as recommended by the National Academy of Engineering (NAE) and the Institute of Medicine (IOM) [25]. Systems engineering emphasises coordination, synchronisation, and integration of complex systems involving personnel, information, materials, and financial resources in order to meet the defined performance objectives [26]. In the context of MR-VSs, this includes patients, allied health professionals operating the diagnostic hub, clinicians interpreting results, software developers, administrative staff, and healthcare managers. The overarching aim is to deliver a fast, efficient, and reliable service that not only allows early detection, risk stratification, monitoring, and management of retinal diseases but also supports F2FC capacity and healthcare system cost-effectiveness. Performance can be assessed using metrics like patient waiting times, diagnostic turnaround times, the accuracy of clinical decisions, patient satisfaction, cost efficiency, and F2FC bring-back rates.

In order to be able to meet these objectives, MR-VSs rely heavily on high-quality, reproducible images, acquired in the shortest time possible. As mentioned in Table 4, Optos has been shown to have a faster acquisition time than Clarus, by a median difference of 10 s [24]. Furthermore, whilst there is no research directly comparing the user-dependency of these UWF imaging modalities, Clarus is likely to be more user-dependent, with higher inter-operator variability. During acquisition of retinal images with Clarus, the technician needs to manually swing the body of the camera to ensure that it is oriented at the right angle. Because a minimum of two image captures is required to produce a 200° montage using Clarus, the technician has a more active role and would need to adjust the lens angle multiple times during the UWF imaging of one eye. This can be especially tricky when imaging the temporal retina, as the technician needs to minimise interference stemming from the patient’s nose through correct orientation. Furthermore, because of the 25 mm working distance, patient movement or poor cooperation could make image acquisition even more difficult and longer. On the other hand, taking a UWF photo with Optos is more straightforward on paper since a sufficient ultra-wide FoV can be achieved with a single capture, with the technician simply pressing the patient’s face against a stationary machine and adjusting the lens using a remote.

These differences in image acquisition and user dependency could explain why a statistically significant difference was found in our cohort with regard to FAF acquisition (*p* = 0.001). Whilst all the Optos patients underwent FAF analysis, 22.4% of the Clarus patients did not. As mentioned above, acquisition with Clarus is more user-dependent, requires a minimum of two image captures for each of the UWF colour image montages and the FAF image montages (amounting to a minimum of four image captures), has a longer acquisition time, and requires good patient cooperation. On the other hand, with Optos, both pseudocolour and FAF images are acquired with a single capture each, making the process faster whilst also decreasing dependency on both the operator and patient. This discrepancy in FAF acquisition shows that there was a deviation from the study protocol for one-fifth of the Clarus patient subgroup. However, this difference did not seem to affect clinical decision-making, since there was no statistically significant greater need to bring patients back for further imaging with Clarus. This indicates that routine FAF image acquisition is not required and should rather be obtained on a case-by-case basis such as for patients followed up for hydroxychloroquine retinopathy (or at risk of developing it). This could be carried out through either set protocols per patient category [7] or through clinician instructions when booking/re-booking an MR-VS.

Other differences include the reported better visualisation of the temporal retina enabled by Optos imaging. One reason for this could be the need to orient the Clarus lens position at a right angle, as mentioned above. The Optos modality’s reported wider depth of focus (due to its ellipsoidal mirror-based confocal scanning laser system) could also play a role as it results in higher-clarity peripheral retinal blood vessels [23,27,28]. Image centration is also important, with the centre of a Clarus 200° montage typically lying slightly nasal to the fovea, resulting in better visualisation of the nasal retina in comparison to the temporal periphery with Clarus [23]. Another reported difference is the higher chance (59.6%) of missing white lesions with Optos in comparison to Clarus (17%), potentially due to the former’s pseudo-colour imaging optics [29].

However, none of these differences seem to exert any significant effects on clinical decision-making in MR-VSs, as found in our study. This is also reflected in other studies, which report that both UWF modalities are comparable in their ability to visualise the retinal periphery for reliable DR grading [28,30].

Of note, Clarus is reported to provide higher theoretical optical resolution, as shown in Table 4. However, both devices produce sufficient image quality at the macula for the diagnosis and management of AMD and DM in a real-world setting, in conjunction with OCT. Furthermore, differences in patient cooperation, technician acquisition techniques, and other intrinsic imaging characteristics of devices likely neutralise any advantage in theoretical resolution during practical clinical assessments.

Even though this is the first real-world comparative study comparing the effect of UWF imaging on outcomes in a MR-VS, involving patients with varied pathologies and of different age groups and ethnicities, it has some limitations. Firstly, we did not assess whether the clinical decisions were correct, which means that no inferences can be made as to whether these different imaging modalities affect clinical decision accuracy. Since our aim was to assess and compare UWF image acquisition with different imaging modalities, as part of the study design, we only included patients with outcomes influenced by UWF imaging and did not assess other clinical parameters (such as VA, IOP, and OCT), which could also contribute to the final outcome in real life. Although data collection took place over a relatively short period and was based on a single institution, it involved six sites throughout the institution, which should have minimised the effect of inter-operator bias.

Although there are important technical differences between the Optos and Clarus imaging modalities, neither was found to be inferior to the other in terms of image quality issues that affected clinical outcomes. Although there are no studies on user-dependence comparability, Clarus is likely to be more user-dependent and have a longer acquisition time due to the need for manual, accurate positioning of the camera body whilst also requiring a minimum of double the images needed with Optos for both a UWF fundal photo and UWF FAF images. Although this does not seem to affect the rate of image quality issues, it does lead to a lower acquisition rate of FAF images with Clarus. However, the MR-VS outcomes were not influenced by this difference, indicating that routine FAF image acquisition is not required in MR-VSs and that these images should instead be obtained when clinically required. Studies directly comparing the user-dependency of these imaging modalities are required, along with studies assessing the effect of inter-observer variability on MR-VS outcomes. In the future, artificial intelligence (AI) could transform the way any MR-VSs are run, with studies showing that AI can overcome interobserver variability and perform better than five out of six experts at predicting disease progression [31].

## Figures and Tables

**Table 1:** Definitions of the Imaging Quality Indices used in this study.

Imaging Quality Index	Definition
Good image quality	Clear and wide field of view that enables confident clinical decision-making
Poor image quality	Generalised unclear view of the fundus, which precludes confident clinical decision-making
Insufficient field of view (FoV)	Area captured in the images does not incorporate a full view up to the vortex veins
Artefact	Focal anomalies due to the imaging process are not thought to be naturally present and are not due to obvious or known clinical pathology

**Table 2:** Baseline characteristics.

Parameter [Valid Cohort; Missing Data]	Total Population [*n* (%)]	Optos Imaging Subgroup [*n* (%)]	Clarus Imaging Subgroup [*n* (%)]	*p*-Value (Optos vs. Clarus)
**Age (years) [*n* = 268]**				*p* = 0.28
Average ± SD (range)	63 ± 17 (16–98)	65 ± 16 (16–96)	63 ± 18 (19–98)	
**Ethnicity [*n* = 267; missing = 1]**				*p* = 0.38
White	73 (27.3%)	45 (29.8%)	28 (24.1%)	
Black	30 (11.2%)	13 (8.6%)	17 (14.7%)	
Asian	73 (27.3%)	43 (28.5%)	30 (25.9%)	
Other	91 (34.1%)	50 (33.1%)	41 (35.3%)	
**Virtual clinic site [*n* = 268]**				*p* = 0.001
Moorfields City Road	137 (51.1%)	114 (75.0%)	23 (19.8%)	
Moorfields Brent Cross	18 (6.7%)	18 (11.8%)	0 (0.0%)	
Moorfields Hoxton	74 (27.6%)	6 (3.9%)	68 (58.6%)	
Moorfields Stratford	22 (8.2%)	1 (0.7%)	21 (18.1%)	
Moorfields Ealing	13 (4.9%)	13 (8.6%)	0 (0.0%)	
Moorfields Northwick Park	4 (1.5%)	0 (0.0%)	4 (3.4%)	
**New versus follow-up patients [*n* = 268]**				*p* = 0.005
New patient	56 (20.9%)	41 (27.0%)	15 (12.9%)	
Follow-up patient	212 (79.1%)	111 (73.0%)	101 (87.1%)	

**Table 3:** Primary diagnosis/reason for review.

Indication [n = 268]	Number (%)	Optos Imaging Subgroup Number (%)	Clarus Imaging Subgroup Number (%)
Diabetic Retinopathy/Maculopathy	85 (31.7%)	42 (27.6%)	43 (37.1%)
Age-related macular degeneration	40 (14.9%)	20 (13.2%)	20 (17.2%)
Central serous chorioretinopathy	37 (13.8%)	19 (12.5%)	18 (15.5%)
Retinal vein occlusion	29 (10.8%)	19 (12.5%)	10 (8.6%)
Myopia	15 (5.6%)	9 (5.9%)	6 (5.2%)
Retinal lesions	10 (3.7%)	7 (4.6%)	3 (2.6%)
Vitreoretinal	6 (2.2%)	5 (3.3%)	1 (0.9%)
Hydroxychloroquine screening	6 (2.2%)	5 (3.3%)	1 (0.9%)
Other	40 (14.9%)	26 (17.1%)	14 (12.1%)

**Table 4:** Technical specifications of Optos and Clarus.

Parameter	Optos California (Optos^®^, Dunfermline, Scotland, UK)	Zeiss Clarus 500 (Carl Zeiss Meditec AG, Oberkochen, Germany)
**Retinal area captured**	Larger (765.6 mm^2^) [20]	Smaller (566.5 mm^2^) [20]
**Field of view (FoV)**	200° single-capture; capacity to create a multiple-image montage in order to create a 220° FoV [21]	133° single-capture; can produce a UWF photo (200°) through a 2-image-montage, with the ability to increase this to a 267° image (six-image montage) [22]
**True colour vs. pseudo-colour**	Pseudo-colour images are produced due to the use of a red-green or red-green-blue laser as a light source [21,22]	Captures true-colour images due to its white light-emitting diode (LED) light source [21,22]
**Optical resolution**	14 microns [21]	7.3 microns [22]
**Image centre**	Fovea [23]	Slightly nasal to the fovea on a 200° montage [23]
**Acquisition technique**	Patient’s face is pressed against a stationary machine, and photos are captured with one click after focusing the lens [21]	25 mm working distance between the front of the lens and the patient’s eye; the technician has to swing the body of the camera right and left to be able to record at least two images for a 200° montage [22]
**Acquisition time**	Shorter acquisition time (median 32 s) [24]	Longer acquisition time (median 42 s) [24]
**Other functions**	Autofluorescence Sensory Red-free (same capture as pseudo-colour) Fluorescein angiography Indocyanide green angiography [21]	Autofluorescence Red-free (separate capture from the colour) Infrared reflectance Live infrared preview (allows accurate alignment in order to decrease artefacts and decrease the need for repeated image captures) [22]
**Other models**	Silverstone, MonacoPro, Daytona	Zeiss Clarus 700 (added fluorescein angiography function)

## Data Availability

Data are available from the corresponding author upon reasonable request.

## Supplementary Materials

The following supporting information can be downloaded at https://www.mdpi.com/article/10.3390/jcm14103270/s1. Supplementary file: Supplementary Material.
