# Supplementary material for: A Comparison of Ultra-Widefield Imaging Quality Obtained with Zeiss Clarus and Optos for Virtual Medical Retina Services
Source: J Clin Med. 2025 May 8;14(10):3270. doi: 10.3390/jcm14103270 (PMC12111814; doi:10.3390/jcm14103270)
Supplement: Supplementary file 1 [file jcm-14-03270-s001.zip › jcm-3621083-supplementary.pdf]

Supplementary Material

With regards to the 6 patients who were reviewed due to vitreoretinal pathology, epiretinal membrane was reported in 4 (66.7%), vitreomacular traction in 2 (33.3%), and posterior vitreous detachment and lamellar macular hole in 1 (16.7%) patient each. 'Retinal lesions' predominantly included retinal naevi and congenital hypertrophy of the retinal pigment epithelium (CHRPE). 'Other' primary diagnoses included macular telangiectasia, sickle cell retinopathy, angioid streak-related choroidal neovascular membrane (CNV), posterior uveitis-related CNV, retinal dystrophies, retinal artery occlusions, post-operative cystoid macular oedema or uveitis, torpedo maculopathy, and idiopathic retinal changes.
